# A novel pathogenic AIP variant associated with familial isolated pituitary adenoma

**DOI:** 10.1007/s11102-026-01672-y

**Published:** 2026-04-20

**Authors:** Valentino Marino Picciola, Anna Crociara, Serena Piacentini, Lucrezia Rossi, Maria Rosaria Ambrosio, Marco Gessi, Antonio d’Amati, Michele Rubini, Maria Chiara Zatelli

**Affiliations:** 1 Section of Endocrinology, Geriatrics and Internal Medicine, Department of Medical Sciences, University of Ferrara, Via Ariosto 35, Ferrara, 44124 Italy; 2 Endocrine Unit, University Hospital S. Anna, Ferrara, 44124 Italy; 3 Mater Olbia Hospital, Olbia, Italy; 4 Department of Life Sciences and Public Health, Section of Anatomic Pathology, Università Cattolica del Sacro Cuore, Rome, Italy; 5 Laboratory of Reproductive Medical Genetics, Department of Neuroscience and Rehabilitation, University of Ferrara, Ferrara, 44121 Italy

**Keywords:** AIP, Familial Isolated Pituitary Adenoma, Pituitary Adenoma, Pituitary adenoma/PitNet, Prolactinoma, Acromegaly

## Abstract

**Purpose:**

Familial Isolated Pituitary Adenoma (FIPA) is a rare autosomal dominant condition associated with germline mutations in the Aryl Hydrocarbon Receptor-Interacting Protein (*AIP*) gene in ~ 20% of the cases. This study aims to characterize a novel *AIP* variant in a Sardinian family through genetic, clinical and immunohistochemical analyses and evaluate its pathogenicity.

**Methods:**

The proband, a 17-year-old girl with an invasive prolactin (PRL)-secreting Pituitary Neuroedocrine Tumor (Pit-NET), also known as a pituitary adenoma, underwent germline *AIP* testing, extended to family members. All variant carriers underwent clinical and radiologic evaluation. Immunohistochemistry for AIP expression was performed on Pituitary adenoma/PitNet tissue from an affected relative. Variant pathogenicity was classified according to guidelines.

**Results:**

A novel heterozygous *AIP* germline variant, c.479del (p.Pro160LeufsTer11 ), was identified in the proband and six relatives. Cabergoline treatment led to PRL levels normalization and tumor shrinkage in the proband. An affected cousin had an aggressive GH-secreting Pituitary adenoma/PitNet requiring multiple treatments. All other carriers had no clinical findings, and their imaging was unremarkable. The variant causes a premature stop codon leading to a truncated protein, lacking the three C-terminal tetratricopeptide repeat domains, essential for AIP protein-protein interactions and tumor suppressor function. Immunohistochemistry showed reduced AIP expression. The variant is classified as pathogenic.

**Conclusion:**

This report expands the variants spectrum of *AIP*-related FIPA, confirming intra-familial phenotypic variability, from asymptomatic carriers to aggressive Pituitary adenoma/PitNet. Early genetic screening and surveillance are essential for timely diagnosis and personalized management.

**Supplementary Information:**

The online version contains supplementary material available at 10.1007/s11102-026-01672-y.

## Introduction

Familial Isolated Pituitary Adenoma (FIPA) syndrome is a clinical condition characterized by the presence of Pituitary adenoma/PitNet, also known as a pituitary adenoma, in at least two members of the same family, in the absence of features indicating other genetic syndromes associated with Pituitary adenoma/PitNet. FIPA accounts for ~ 2% of all diagnosed Pituitary adenoma/PitNet cases [1]. This condition is associated in ~ 20% of the cases with germline mutations in the Aryl Hydrocarbon Receptor-Interacting Protein (*AIP*) tumor suppressor gene. These mutations follow an autosomal dominant inheritance pattern and display incomplete penetrance (20–23%) [2]. The *AIP* gene encodes a 330 amino acid (aa) cytoplasmic protein (~ 37 kDa) acting as a ligand-activated transcriptional co-regulator. Structurally, AIP contains an N-terminal FK506-binding protein (FKBP)-like domain (aa 31–121), and a C-terminal region comprising three tetratricopeptide repeat (TPR) domains (TPR1: aa 179–212; TPR2: aa 231–264; TPR3: aa 265–298), each consisting of a 34 aa alpha-helical motif. These TPR domains are involved in protein–protein interactions, mediating AIP function in various cellular pathways [3]. Most pathogenic *AIP* variants result in the loss of the C-terminal portion or of the entire protein, ultimately leading to loss of function. Missense mutations and insertions, affecting protein folding and stability, promoting proteasomal degradation, are less frequent [2]. Germline *AIP* variants are typically suspected in young patients presenting with invasive growth hormone (GH) and/or prolactin (PRL) secreting Pituitary adenoma/PitNets, particularly when there is a family history. Affected individuals tend to share a distinctive clinical profile, including early age at onset, large tumor size, and aggressive behavior [1, 2, 4, 5]. Timely genetic diagnosis is critical not only for prognosis and tailored treatment, but also for the identification of asymptomatic carriers within the family [4, 5]. This approach supports targeted follow-up and proactive clinical management, ultimately aiming to improve patient outcomes. Here, we describe a novel *AIP* variant identified in a large Sardinian family, where the proband is a young female patient diagnosed with a PRL-secreting PA.

## Materials, subjects and methods

### Patients

The proband is a 17-year-old girl referred for primary amenorrhea and headache, with a family history of Pituitary adenoma/PitNet in the maternal side: two cousins had been diagnosed before the age of 40 years with a GH-secreting Pituitary adenoma/PitNet and a non-functioning (NF) Pituitary adenoma/PitNet, respectively. Patient’s clinical evaluation was unremarkable. Blood tests showed high plasma PRL levels (2209 ng/ml; normal values < 26.5) (Supplementary Table 1), in the absence of macroprolactinemia. Pituitary Magnetic Resonance Imaging (MRI) showed a Pituitary adenoma/PitNet of 2 × 2 × 1.9 cm, with sellar widening, suprasellar extension, slight optic chiasm compression, left cavernous sinus invasion (grade IV according to the Zurich Pituitary Score and grade 4 according to Knosp). Visual field was normal. Medical therapy with 1 mg/week cabergoline determined a reduction in PRL levels (109.7 ng/ml) and menarche onset. After 12 months the Pituitary adenoma/PitNet significantly shrunk (1.1 × 1.7 × 1.6 cm), with persistence of left cavernous sinus invasion. After 24 months, PRL levels were normal (17.8 ng/ml) with a further Pituitary adenoma/PitNet volume reduction (1.0 × 1.1 × 1.6 cm) (Supplementary Fig. 1). At each clinical follow-up IGF-1 levels were always in the normal range and there were no signs and symptoms suggestive of GH excess.

**Fig. 1:**
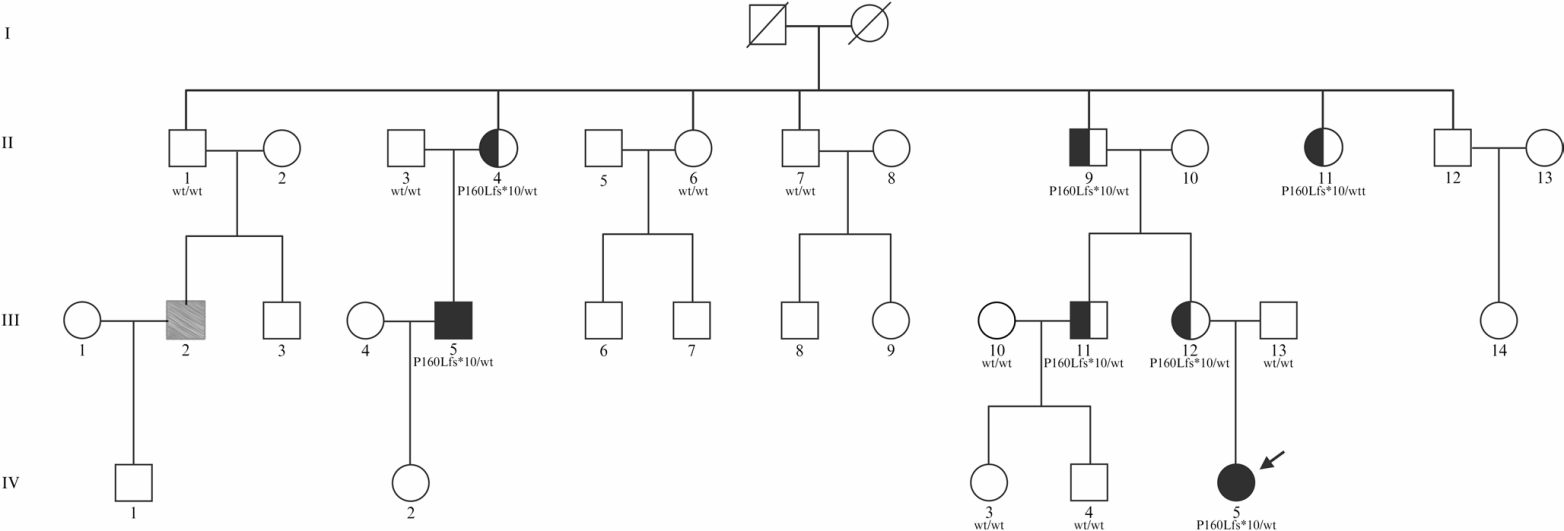
Pedigree of investigated family. Square symbols: male, circles: female. Deceased individuals are indicated by a crossed-out symbol. Genotypes for the c.479del variant (p.Pro160LeufsTer11) are shown for all subjects for whom a genomic DNA sample was available. Filled symbols represent family members with Pituitary adenoma/PitNet. Proband (IV 5) is indicated by the arrow. Filled symbols represent affected carriers with Pituitary adenoma/PitNet. Half-filled symbols represent unaffected carriers. The grey-filled symbol indicates a carrier affected by non-functioning Pituitary adenoma/PitNet. Created in https://BioRender.com”

Genetic investigation for germline *MEN1* and *CDKN1B* pathogenic variants was negative. Therefore, *AIP* genetic testing was performed. Family members were investigated and 1 was found to carry a Pituitary adenoma/PitNet in association with the reported novel *AIP* variant at germline level (Fig. 1). The patient remains under continuous clinical and imaging follow-up.

### DNA isolation and sequencing

Genomic DNA (gDNA) from each patient included in the study was isolated as previously described [6]. Subsequently, *AIP* exons were amplified by polymerase chain reaction (PCR) using the Taq DNA Polymerase kit (Thermo Fisher Scientific, Milano, Italy) for exons 2, 4, 5 and 6 and the Taq DNA Polymerase kit (Merck KGaA, Darmstadt, Germany) for exons 1 and 3. The oligonucleotides sequences (Integrated DNA Technologies, Iowa, USA) used in the amplification step and the PCR protocols used for amplification of each exon on the Veriti Thermal Cycler instrument (Applied Biosystems, Massachusetts, USA) are described in Supplementary Table 2. Subsequently, PCR products were purified with the QIAquick PCR Purification Kit (QIAGEN, Milano Italy) on the QIAcube system (QIAGEN) and submitted to direct sequencing with the same primers and the BigDye Terminator v3.1 Cycle Sequencing Kit (Thermo Fisher Scientific). After standard purification, sequencing reaction products were loaded onto the 3500 Dx Series Genetic Analyzer CE-IVD instrument (Applied Biosystems) by using the BDx Rapid Seq Assay POP7 running protocol (Applied Biosystems) and the Sequencing Analysis v.5.4 software. DNA sequences were compared with the wild-type sequence of the same gene region. The transcript NM_003977.4 was used as reference for the cDNA sequence, and NP_003968.3 was used as the reference for the corresponding protein sequence [7].

### Prediction of the impact of the *AIP* sequence variant on AIP protein structure

The UniProt knowledgebase (https://www.uniprot.org/) [8] was used to predict the impact of the identified AIP gene variant on AIP protein structure. This platform visualizes nucleotide and amino acid sequences as well as protein crystallographic structure. The Human Protein Atlas resource (www.proteinatlas.org) was used to illustrate the AIP protein’s secondary structure.

### Immunohistochemistry

Immunohistochemistry (IHC) was performed on tissues obtained from the Pituitary adenoma/PitNet of a family member carrying a GH-secreting Pituitary adenoma/PitNet (Fig. 1, subject III-5) and the described germline *AIP* variant. This patient underwent surgery for therapeutic purposes at 37 years of age, according to current guidelines [9–11]. Two specific anti AIP-antibodies (Ab) (Thermo Fisher Scientific) were employed for IHC: the first (#PA5-29862, Rabbit, Polyclonal) recognizes AIP central portion (aa 37 to 294) and the second (#PA5-18715, Goat, Polyclonal) recognizes AIP C-terminal portion (aa 313 to 323), using normal human placenta and normal pituitary gland tissue present at the periphery of the sample as positive control. IHC was performed on a Leica BOND III platform following the manufacturer’s indication for antigen unmasking at high pH and enzyme digestion.

### Extended next-generation sequencing (NGS) analysis in variant carriers

All individuals carrying the germline *AIP* variant underwent targeted NGS of a panel of pituitary tumor–predisposition genes (Supplementary Table 3) to provide external validation of the AIP finding and to assess the presence of additional germline pathogenic variants.

### Variant interpretation and classification

Variant classification was performed according to the standards and guidelines for the interpretation of sequence variants established by the American College of Medical Genetics and Genomics (ACMG) and the Association for Molecular Pathology (AMP) in 2015. This framework evaluates multiple lines of evidence to assign variants to one of five categories: pathogenic, likely pathogenic, uncertain significance, likely benign, or benign [12].

## Results

### Family pedigree

A heterozygous variant was found in the proband gDNA in exon 4 of the *AIP* gene: c.479del (p.Pro160LeufsTer11 ). The proband underwent genetic testing at 17 years of age, immediately after the diagnosis of a pituitary adenoma. Once the *AIP* variant was identified, genetic analysis was extended to the family, revealing the same variant in six consanguineous relatives (Fig. 1).

Among them, the proband’s cousin (subject III-5), a 40-year-old male, had already been diagnosed with a GH-secreting Pituitary adenoma/PitNet prior to the genetic test at 35 years. Acromegaly was suspected due to characteristic acromegalic facial features, while height and weight were within the normal range (177 cm and 79 kg). Hormonal evaluation revealed markedly elevated IGF-1 levels (900 ng/mL; reference range 122–524 ng/mL) and GH hypersecretion was confirmed by an OGTT. The remaining pituitary function was normal. Pituitary MRI demonstrated a 12 × 13 × 18 mm Pituitary adenoma/PitNet with left cavernous sinus invasion and encasement of the internal carotid artery (Knosp grade IV). At 37 years of age, the patient underwent transsphenoidal surgery, which resulted in incomplete tumor resection. Postoperative evaluation showed persistent elevated IGF-1 levels (451 ng/mL) and lack of GH suppression on OGTT (nadir 2.55 µg/L). Postoperative MRI confirmed residual tumor, and no further surgical intervention was attempted due to the challenging site of the residual disease. Subsequently, medical therapy with a first generation long-acting somatostatin analog (Lanreotide 120 mg every 28 days) was started and continues to date. He also underwent Gamma Knife radiosurgery. Despite these interventions, residual tumor tissue persists. At the last follow-up, IGF-1 levels were 127 ng/mL and MRI showed a residual 0.3 cm lesion in the right paramedian adenohypophysis.

Genomic DNA was isolated from the tumor tissue but was markedly degraded, precluding loss of heterozygosity (LOH) analysis of the *AIP* gene. The other five members underwent clinical and radiologic screening at the same age at which the mutation was detected: III-12 at 44 years, III-11 at 47 years, II-4 at 63 years, II-9 at 72 years, and II-11 at 70 years. In each of them, pituitary MRI and clinical evaluation were unremarkable, with no signs or symptoms consistent with Pituitary adenoma/PitNet at the time of assessment (Supplementary Table 4). All unaffected variant carriers continue to undergo periodic clinical and imaging follow-up, as previously suggested [1]. Subject III-2 (see Fig. 1) had previously undergone surgical treatment for a NF Pituitary adenoma/PitNet at the age of 40 years, but further clinical and histopathological details were not available. He denied genetic testing; however, his father (II-1) is wild-type for the *AIP* variant.

### Predicted structural consequences of the *AIP* c.479del variant

The identified nucleotide variant c.479del consists of a Cytosine at position 479 within exon 4 of the *AIP* gene, causing a frameshift that causes the substitution of Proline at codon 160 with Leucine, and a stop codon in the new reading frame 10 codons after the affected Pro160 amino acid residue (p.Pro160LeufsTer11 ) (Table 1).

**Table 1:** Amino acid modifications

Codon number	160	161	162	163	164	165	166	167	168	169	170
Wild Type	**Pro**	Gly	Thr	Tyr	Gln	Gln	Asp	Pro	Trp	Ala	Met
Reported variant	**Leu**	Ala	Arg	Thr	Ser	Arg	Thr	His	Gly	Pro	**STOP**

Comparison of wild-type and mutant AIP amino acid sequences showing a frameshift at codon 160 resulting in a truncated protein

**Fig. 2:**
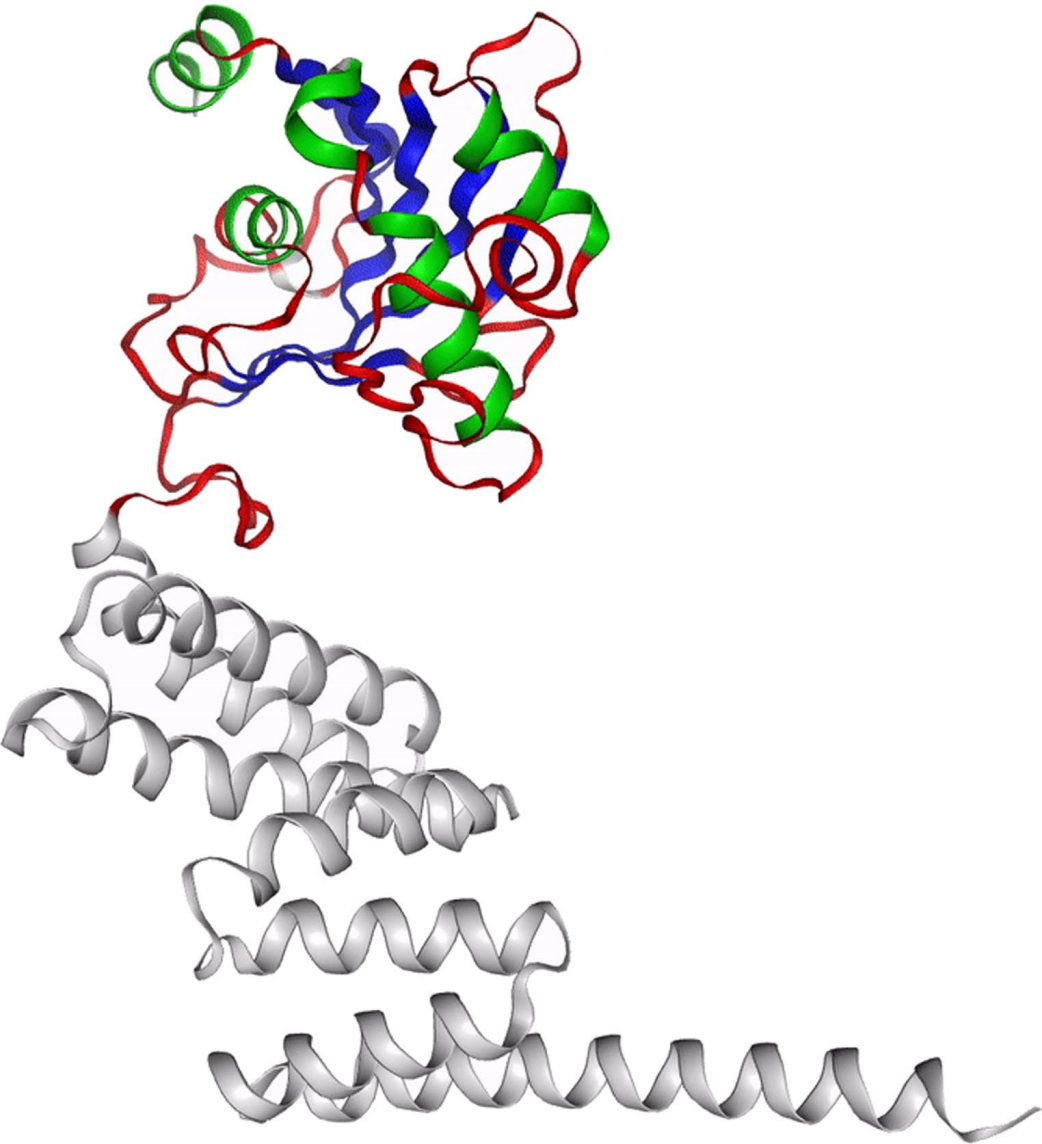
Secondary structure of the AIP protein. The grey segment highlights the C-terminal region lost due to the sequence variant. Created in https://www.proteinatlas.org

The insertion of this new stop codon results in a truncated AIP protein that retains the 169 aa N-terminal peptide sequence, including the peptidyl-prolyl cis-trans isomerase (PPIase) FKBP-type domain (aa 31–121) while completely lacks the three TPR domains in the C-terminal part of the protein, which are essential for protein-protein interaction and tumor suppressor function (Fig. 2).

### IHC findings

**Fig. 3:**
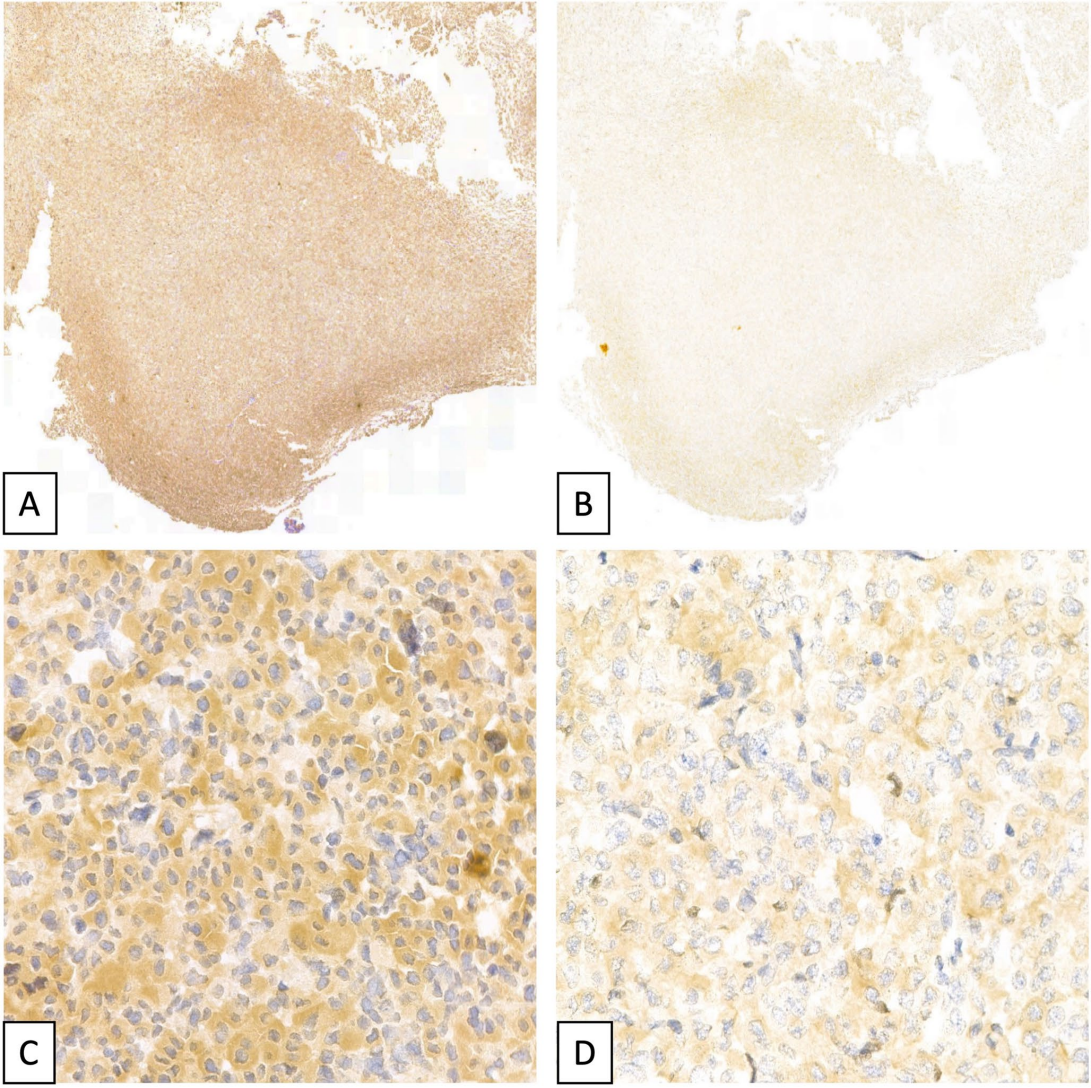
IHC results in the mutated GH-secreting Pituitary adenoma/PitNet. Staining for AIP protein with anti-AIP rabbit antibody, recognizing AIP central portion, in the GH-secreting Pituitary adenoma/PitNet of the carrier relative (subject III5) (A: 5x magnification; C 100x magnification). Staining for AIP protein with anti-AIP goat antibody, recognizing AIP C-terminal portion, in the GH-secreting Pituitary adenoma/PitNet of the carrier relative (subject III5) (B: 5x magnification; D: 100x magnification)

Diffuse and intense staining for the antibody recognizing the central part of the AIP protein was observed in the peripheral rim of non-neoplastic tissue and in tumor cells of the investigated sample (Fig. 3A and C). A reduction in intensity was observed for the antibody directed against the AIP C-terminal (Fig. 3B and D), potentially consistent with the reduced protein expression expected from the heterozygous *AIP* gene status of this patient. Supplementary Fig. 2 shows the IHC results for AIP staining with the antibody recognizing AIP central part and the antibody directed against the AIP C-terminal in an AIP wild type GH-secreting Pituitary adenoma/PitNet and in a positive control (human placenta).

### NGS

NGS analysis performed in all germline carriers confirmed the presence of the AIP c.479del (p.Pro160LeufsTer11 ) variant and excluded additional pathogenic or likely pathogenic variants in other pituitary tumor–predisposition genes included in the panel.

### Variant classification

Based on the ACMG/AMP 2015 criteria [12], the *AIP* variant c.479del (p.Pro160LeufsTer11) is classified as pathogenic. This classification is supported by the variant type (frameshift with early stop codon; PVS1), its absence in population databases (PM2), and familial co-segregation with disease (PP1), as detailed in Table 2.

**Table 2:** Variant classification according to ACMG/AMP 2015 criteria

ACMG/AMP Criterion	Assessment for AIP c.479del (*p*.Pro160LeufsTer11)
PVS1 (Very Strong)	This frameshift variant results in a truncated AIP protein that lacks all three TPR domains which are essential for AIP’s tumor suppressor function. The predicted LOF effect is a well-established mechanism in AIP-related disease.
Null variant (nonsense, frameshift, canonical ± 1/2 splice site) in a gene where LOF is a known disease mechanism.	
PM2 (Moderate)	The variant is absent from large population databases such as gnomAD, 1000 Genomes Project and ClinVar. Its absence supports rarity. It likely represents a private or founder mutation in this Sardinian family.
Absent or extremely rare in population databases.	
PP1 (Supporting)	This variant co-segregates with disease in two affected relatives (the proband and her cousin), both diagnosed with PitNETs of different types (PRL- and GH-secreting).
Co-segregation with disease in multiple affected family members.	

*PVS* Pathogenic Very Strong, *PM* Pathogenic Moderate, *PP* Pathogenic Supporting, *LOF* Loss of Function

The AIP c.479delC variant (p.Pro160LeufsTer10) meets ACMG/AMP 2015 standards for a pathogenic classification

## Discussion

The c.479del (p.Pro160LeufsTer11 ) variant identified in this FIPA family is predicted to profoundly disrupt AIP interaction with several cellular partners, possibly hampering its tumor suppressor function. This variant encodes for a truncated AIP protein, which aligns with numerous studies demonstrating that about 75% of pathogenic *AIP* variants are truncating. These variants typically occur in the protein’s C-terminal region, disrupting the function of the TPR domains and likely contributing to the development of Pit-NET [2], also known as a pituitary adenoma. Under physiological conditions, AIP interacts with the phosphodiesterases PDE4A4 and PDE4A5 to facilitate cyclic AMP (cAMP) degradation. The loss of TPR domains impairs this interaction, leading to intracellular cAMP accumulation, which, in turn, promotes mitogenic signaling and tumorigenesis [2, 13]. Furthermore, AIP is involved in the expression regulation of zinc finger protein regulating apoptosis and cell cycle arrest 1 (*ZAC1*), a tumor suppressor gene. The AIP-ZAC1 interaction importantly influences pituitary cell function and is particularly relevant to SSA efficacy [14]. Reduced ZAC1 expression has been observed in *AIP*-mutated Pituitary adenoma/PitNets and has been associated with increased proliferation and decreased sensitivity to SSA [2]. These evidences support the hypothesis that the *AIP* frameshift variant detected in the studied family is associated with Pituitary adenoma/PitNet development.

The absence of this variant in global databases (Table 2) suggests that it may represent a private or founder mutation within this Sardinian family. Sardinia is a relatively genetically isolated population, and such geographic and demographic features may contribute to the presence of rare genetic variants. This context may explain the current lack of reports for this specific variant and further supports the need for continued surveillance and characterization of novel *AIP* variants in underrepresented populations.

Clinically, the identification of this rare variant in a family with multiple affected members provides an opportunity to examine its phenotypic consequences. FIPA can be classified as homogeneous, when all affected individuals within a family develop the same type of Pituitary adenoma/PitNet, or heterogeneous, when different tumor subtypes occur among family members [1]. The family presented here represents an example of a heterogeneous FIPA, with the proband affected by a PRL-secreting Pituitary adenoma/PitNet and her cousin by a GH-secreting Pituitary adenoma/PitNet. This phenotypic heterogeneity aligns with previous reports describing that *AIP*-related tumors are most commonly GH-secreting, PRL-secreting, or mixed GH/PRL-secreting Pituitary adenoma/PitNets [1, 2].

In our family, genetic testing identified a novel heterozygous *AIP* variant c.479del in seven individuals. Of these, only two had clinically evident Pituitary adenoma/PitNets, underscoring the well-documented incomplete penetrance of *AIP* mutations [1, 2]. Notably, the remaining five variant carriers were asymptomatic, with normal endocrine profiles and unremarkable pituitary MRIs at the time of evaluation. This finding highlights the clinical challenge posed by incomplete penetrance in the management and surveillance of unaffected mutation carriers. While regular endocrine and radiological follow-up is prudent, over-medicalization must be avoided.

Our findings highlight the importance of targeted genetic evaluation in Pit-NETs. Testing for *AIP* is indicated in patients with a family history of Pituitary adenoma/PitNets, early-onset disease, or clinically aggressive macroadenomas [2, 15–17]. Additionally, GH- or PRL-secreting tumours resistant to medical or surgical treatment, especially when large or invasive, should also prompt *AIP* testing [16, 19]. The observed intra-familial variability, both in tumor type and clinical course, despite a shared germline variant, suggests the influence of additional modifying factors, which may include genetic, epigenetic, or environmental components.

Indeed, one family member (Subject III2, Fig. 1) presented with a clinically diagnosed NF Pituitary adenoma/PitNet, but refused genetic testing. Notably, the father, who tested negative for the AIP variant, also had a negative result on a multi-gene panel screening for other Pituitary adenoma/PitNet-associated mutations, whereas the mother was not tested. Therefore, the genetic status of III2 remains unknown. The occurrence of a Pituitary adenoma/PitNet in this individual accounts for a phenocopy in this FIPA family; it may represent a coincidental finding, given that incidental pituitary lesions have been reported in approximately 10–38% of the general population [20], or may indicate the presence of additional factors contributing to Pituitary adenoma/PitNet development in this family.

Even among affected individuals, the expression of disease differed markedly: the proband presented with a PRL-secreting Pituitary adenoma/PitNet at age 17, with a favorable biochemical and radiological response to dopamine agonist therapy. Her cousin, diagnosed with a GH-secreting Pituitary adenoma/PitNet at 35 years, required transsphenoidal surgery followed by Gamma Knife radiosurgery and ongoing SSA therapy, reflecting a more aggressive tumor behavior. Such clinical variability in the context of *AIP*-related Pituitary adenoma/PitNet underlines the importance of individualized clinical management.

The proband young age at diagnosis is consistent with prior findings showing that Pituitary adenoma/PitNet patients harboring *AIP* pathogenic variants typically present earlier than those without [1, 2, 15]. Moreover, both affected individuals in our report had macroadenomas, a common feature in *AIP*-mutated Pituitary adenoma/PitNets [15, 16, 18]. Tumor invasiveness was also evident in both cases, with cavernous sinus invasion observed at MRI, in line with previous studies [16, 18].

AIP-mutated PRL-secreting Pituitary adenoma/PitNet are often reported to exhibit resistance to dopamine agonists (DA) [16], while in the reported case cabergoline therapy determined a progressive tumor shrinkage and serum PRL levels normalization. These findings suggest that DA sensitivity may be preserved in some AIP-related PRL-secreting Pituitary adenoma/PitNet, as also supported by a recent comparative study reporting > 50% tumor shrinkage in 58.3% of *AIP* related prolactinomas, compared with only 4.2% in genetically negative DA resistant cases [21]. Nevertheless, long-term follow-up is essential to monitor for recurrence or secondary resistance.

GH-secreting Pituitary adenoma/PitNets associated with *AIP* pathogenic variants are typically aggressive and less responsive to SSA. These patients often require multiple neurosurgical procedures and radiotherapy [16]. The clinical course of the proband’s cousin, who underwent surgery and Gamma Knife radiosurgery and has residual disease requiring treatment with SSA, is in line with previous findings.

Under physiological conditions, AIP is predominantly found in somatotropic and lactotropic cells. In Pituitary adenoma/PitNets, however, its expression extends to all tumor cell types, with particularly high levels observed in somatotropinomas and NF Pituitary adenoma/PitNets [2]. Consistently, IHC analyses have confirmed that AIP immunoreactivity is highest in somatotroph and NF adenomas. However, within somatotroph tumors, invasive forms display significantly lower AIP expression compared to non-invasive counterparts, suggesting that reduced AIP expression may contribute to the aggressive behavior of somatotrophinomas [19].

In the reported family, at least one patient (III-5) carried a GH-secreting Pituitary adenoma/PitNet, which showed at IHC a relatively reduced AIP expression, consistent with the *AIP* variant heterozygous status of the patient. This finding should be considered with caution, since IHC is a qualitative rather than quantitative technique to assess protein expression. Indeed, several variables may influence the IHC performance, including tissue fixation and antibody variable affinity. Nonetheless, the present case was tested for two antigens on the same tissue specimen, normalizing tissue-related problems. In addition, we adopted all possible measure to reduce technical bias including the use of the same platform for both tests with the same stringent unmasking and revelation steps, as well as positive and negative controls.

Although tumor tissue was available for molecular analyses, the isolated DNA was markedly degraded, preventing reliable genomic analysis and LOH assessment of the AIP gene. Therefore, we cannot establish whether somatic loss of the wild-type allele contributed to the reduced AIP expression observed in tumor tissue.

In line with ACMG/AMP 2015 guidelines [12], the c.479del (p.Pro160LeufsTer11 ) variant meets multiple criteria for pathogenicity: predicted loss of function consistent with the gene’s disease mechanism (PVS1), absence from population databases (PM2), and segregation with disease in affected family members (PP1). Although novel, its predicted disruption of all three TPR domains aligns with established pathogenic mechanisms in AIP-related Pituitary adenoma/PitNets, reinforcing its clinical significance.

## Conclusion

Although the c.479del variant has not been previously reported in public mutation databases, the consequent molecular modifications and the predicted loss of key functional domains provide strong evidence for its pathogenic role. Taken together, our findings describe a novel *AIP* variant within a family affected by heterogeneous FIPA. Notably, the occurrence of an NF-Pituitary adenoma/PitNet in a likely non-carrier individual represents a phenocopy within the family, further underscoring the clinical and genetic complexity of FIPA. This report highlights the phenotypic variability and incomplete penetrance commonly observed in *AIP*-related disease, reinforcing the importance of an integrated approach combining genetic, biochemical, and radiological assessments for early diagnosis, risk stratification, and personalized management of predisposed individuals.

## Supplementary Information

Below is the link to the electronic supplementary material.

Supplementary Material 1 (PDF 617 KB)

Supplementary Material 2 (PDF 5.12 MB)

Supplementary Material 3 (PDF 54 KB)

Supplementary Material 4 (PDF 177 KB)

Supplementary Material 5 (PDF 489 KB)

Supplementary Material 6 (PDF 471 KB)

## Data Availability

Data are available on reasonable request.
