# Supplementary material for: A novel pathogenic AIP variant associated with familial isolated pituitary adenoma
Source: Pituitary. 2026 Apr 20;29(3):75. doi: 10.1007/s11102-026-01672-y (PMC13095975; doi:10.1007/s11102-026-01672-y)
Supplement: Supplementary file 1 — Supplementary Material 1 (PDF 617 KB) [file 11102_2026_1672_MOESM1_ESM.pdf]

Pituitary

A novel pathogenic AIP variant associated with Familial Isolated Pituitary Adenoma

Valentino Marino Picciola[1*], Anna Crociara[2*], Serena Piacentini[3], Lucrezia Rossi[1], Maria Rosaria Ambrosio[1-2], Marco Gessi[4], Antonio d'Amati[4], Michele Rubini[5], Maria Chiara Zatelli[1-2]

* These Authors equally contributed to the work.

Affiliations

[1]Section of Endocrinology, Geriatrics and Internal Medicine, Department of Medical Sciences, University of Ferrara, 44124 Ferrara, ITALY

[2]Endocrine Unit, University Hospital S. Anna, 44124 Ferrara, ITALY

[3]Mater Olbia Hospital, Olbia, ITALY

[4]Department of Life Sciences and Public Health, Section of Anatomic Pathology, Università Cattolica del Sacro Cuore, Rome, Italy.

[5]Laboratory of Reproductive Medical Genetics, Department of Neuroscience and Rehabilitation, University of Ferrara, 44121 Ferrara, ITALY

Corresponding Author

Prof. Maria Chiara Zatelli

E-mail: ztlmch@unife.it

Supplementary Figure 1: T1-weighted Fast Spin Echo MRI of the proband PitNET at first observation and at last follow-up.

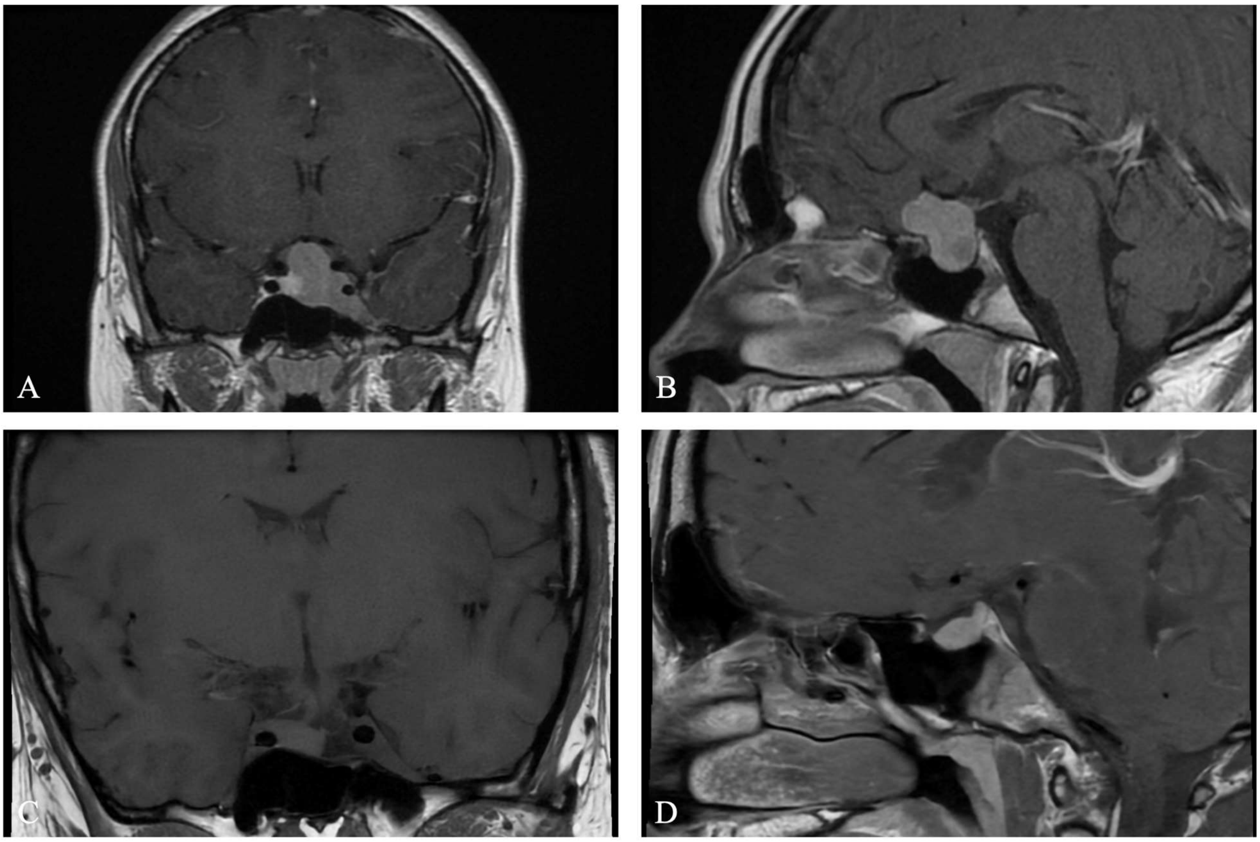

A–B: Coronal and sagittal sections showing a PitNET with marked sellar enlargement, extension into the optic chiasm region, suprasellar cistern, and cavernous sinus invasion.

C–D: Coronal and sagittal follow-up images showing a reduction in tumor volume.
