# Supplementary material for: A novel pathogenic AIP variant associated with familial isolated pituitary adenoma
Source: Pituitary. 2026 Apr 20;29(3):75. doi: 10.1007/s11102-026-01672-y (PMC13095975; doi:10.1007/s11102-026-01672-y)
Supplement: Supplementary file 3 — Supplementary Material 3 (PDF 54 KB) [file 11102_2026_1672_MOESM3_ESM.pdf]

Pituitary

A novel pathogenic AIP variant associated with Familial Isolated Pituitary Adenoma

Valentino Marino Picciola[1*], Anna Crociara[2*], Serena Piacentini[3], Lucrezia Rossi[1], Maria Rosaria Ambrosio[1-2], Marco Gessi[4], Antonio d'Amati[4], Michele Rubini[5], Maria Chiara Zatelli[1-2]

* These Authors equally contributed to the work.

Affiliations

[1]Section of Endocrinology, Geriatrics and Internal Medicine, Department of Medical Sciences, University of Ferrara, 44124 Ferrara, ITALY

[2]Endocrine Unit, University Hospital S. Anna, 44124 Ferrara, ITALY

[3]Mater Olbia Hospital, Olbia, ITALY

[4]Department of Life Sciences and Public Health, Section of Anatomic Pathology, Università Cattolica del Sacro Cuore, Rome, Italy.

[5]Laboratory of Reproductive Medical Genetics, Department of Neuroscience and Rehabilitation, University of Ferrara, 44121 Ferrara, ITALY

Corresponding Author

Prof. Maria Chiara Zatelli

E-mail: ztlmch@unife.it

Supplementary Table 1: Proband hormonal levels at first observation.

Test	Results	Reference Limits
PRL	2209	5.18-26.5 ng/ml
Cortisol	10	3.70-19.4 µg/dl
ACTH	35.42	4.70-48.8 pg/ml
FSH	1.62	3.9-8.8 mU/ml
LH	0.18	Premenopausal women (mIU/mL):<br>Follicular phase: 1.80 - 11.78<br>Ovulatory phase: 7.59 - 89.08<br>Luteal phase: 0.56 - 14.0
Estradiol	< 24	Premenopausal women (pg/mL):<br>Follicular phase: 21 - 251<br>Ovulatory phase: 38 - 649<br>Luteal phase: 21 – 312
Progesterone	< 0.50	Premenopausal women (ng/ml):<br>Follicular phase: 3.03 - 8.08<br>Mid-cycle (ovulatory) phase: 2.55 -16.69<br>Luteal phase: 1.38 - 5.47
Testosterone	1.69	0.48-1.85 nmol/L
DHEA-S	308.20	61.20-493.6 µg/dl
IGF1	254.2	122-524 ng/ml
TSH	1.08	0.35-4.94 µU/ml
FT4	0.81	0.70-1.48 pg/dL
Na	137	136-145 mEq/l
K	3.8	3.5-5.3 mEq/l
Glucose	79	70-110 mm/dl
