# Supplementary material for: A novel pathogenic AIP variant associated with familial isolated pituitary adenoma
Source: Pituitary. 2026 Apr 20;29(3):75. doi: 10.1007/s11102-026-01672-y (PMC13095975; doi:10.1007/s11102-026-01672-y)
Supplement: Supplementary file 4 — Supplementary Material 4 (PDF 177 KB) [file 11102_2026_1672_MOESM4_ESM.pdf]

Pituitary

# A novel pathogenic AIP variant associated with Familial Isolated Pituitary Adenoma

Valentino Marino Picciola[1*], Anna Crociara[2*], Serena Piacentini[3], Lucrezia Rossi[1], Maria Rosaria Ambrosio[1-2], Marco Gessi[4], Antonio d'Amati[4], Michele Rubini[5], Maria Chiara Zatelli[1-2]

* These Authors equally contributed to the work.

**Affiliations**

[1]Section of Endocrinology, Geriatrics and Internal Medicine, Department of Medical Sciences, University of Ferrara, 44124 Ferrara, ITALY

[2]Endocrine Unit, University Hospital S. Anna, 44124 Ferrara, ITALY

[3]Mater Olbia Hospital, Olbia, ITALY

[4]Department of Life Sciences and Public Health, Section of Anatomic Pathology, Università Cattolica del Sacro Cuore, Rome, Italy.

[5]Laboratory of Reproductive Medical Genetics, Department of Neuroscience and Rehabilitation, University of Ferrara, 44121 Ferrara, ITALY

**Corresponding Author**

Prof. Maria Chiara Zatelli

E-mail: ztlmch@unife.it

**Supplementary Table 2: Exon-specific primers with nucleotide sequence and amplification protocols.**

| **Cycles** / **Exon** | **Primer sequence (5’ -> 3’)** | **Denaturation** | | **Annealing** | **Extension** | |
|---|---|---|---|---|---|---|
| | | Temperature<br>Time | Temperature<br>Time | Temperature<br>Time | Temperature<br>Time | Temperature<br>Time |
| **Ex 1 (F)** | CCG AGA CAT TCC TAG GCT CC | 95 °C | 95 °C | 63 °C | 72 °C | 72 °C |
| **Ex 1 (R)** | CTC TCG CCT AAG GCC TCC | 3' | 30" | 45" | 30" | 4' |
| **Ex 2 (F)** | GGA CTG GAC TTC TCC TTG GG | 95 °C | 95 °C | 67 °C | 72 °C | 72 °C |
| **Ex 2 (R)** | GTC TAG CAG AGG GTG GAG GG | 3' | 30" | 45" | 45" | 4' |
| **Ex 3 (F)** | GAT GGT GGT GGG GAA GG | 95 °C | 95 °C | 64 °C | 72 °C | 72 °C |
| **Ex 3 (R)** | ACC CCT GGG TGG ACA GG | 3' | 30" | 1' | 30" | 4' |
| **Ex 4-5 (F)** | ATG TGG GTC AGG TCT GCT G | 94 °C | 94 °C | 65 °C | 72 °C | 72 °C |
| **Ex 4-5 (R)** | AAA GCC TAG GTC TTG ACC CC | 3' | 30" | 45" | 45" | 4' |
| **Ex 6 (F)** | GAC ATG AGG GCA GGC AGC T | 95 °C | 95 °C | 66 °C | 72 °C | 72 °C |
| **Ex 6 (R)** | AGG TGA TGA CCC GGC TCT C | 3' | 30" | 1' | 45" | 7' |
| | | *1 Cycle* | *35 Cycles* | | | *1 Cycle* |

(Ex): Exon; (F): Forward; (R): Reverse.
