# Supplementary material for: A novel pathogenic AIP variant associated with familial isolated pituitary adenoma
Source: Pituitary. 2026 Apr 20;29(3):75. doi: 10.1007/s11102-026-01672-y (PMC13095975; doi:10.1007/s11102-026-01672-y)
Supplement: Supplementary file 5 — Supplementary Material 5 (PDF 489 KB) [file 11102_2026_1672_MOESM5_ESM.pdf]

Pituitary

A novel pathogenic AIP variant associated with Familial Isolated Pituitary Adenoma

Valentino Marino Picciola[1*], Anna Crociara[2*], Serena Piacentini[3], Lucrezia Rossi[1], Maria Rosaria Ambrosio[1-2], Marco Gessi[4], Antonio d'Amati[4], Michele Rubini[5], Maria Chiara Zatelli[1-2]

* These Authors equally contributed to the work.

Affiliations

[1]Section of Endocrinology, Geriatrics and Internal Medicine, Department of Medical Sciences, University of Ferrara, 44124 Ferrara, ITALY

[2]Endocrine Unit, University Hospital S. Anna, 44124 Ferrara, ITALY

[3]Mater Olbia Hospital, Olbia, ITALY

[4]Department of Life Sciences and Public Health, Section of Anatomic Pathology, Università Cattolica del Sacro Cuore, Rome, Italy.

[5]Laboratory of Reproductive Medical Genetics, Department of Neuroscience and Rehabilitation, University of Ferrara, 44121 Ferrara, ITALY

Corresponding Author

Prof. Maria Chiara Zatelli

E-mail: ztlmch@unife.it

Supplementary Table 3: Next-generation sequencing analysis.

Parallel massive sequencing (NGS) uses the MiSeq Reagent kit v3H600 cycles on a MiSeq platform (Illumina), Bioinformatic analysis has been performed with the pipeline DRAGEN Enrichment App (Illumina) v.3.10.4, DECoN'v.1.0.1. Alignment and variant calling versus reference genome GRCh37/hg19.
The test has been performed by the kit Library prep QiaSeq FX DNA library (Qiagen) and capture enrichment with IDT® probes for the coding regions and the intron-exon junctions (±25 bases) of the following genes:

**Gene**	**Transcript (RefSeq)**
AIP	NM_003977.3
AP2S1	NM_004069.4
APC °	NM_000038.6
ARMC5	NM_001105247.1
ATM	NM_000051.4
AXIN2	NM_004655.4
BAP1	NM_004656.3
BMPR1A	NM_004656.3
BRCA1	NM_007294.4
BRCA2	NM_000059.4
BRK1 *	NM_018462.4
CASR	NM_000388.3
CDC73	NM_024529.4
CDH1	NM_004360.5
CDKN1A	NM_078467.2
CDKN1B	NM_004064.4
CDKN2B	NM_004936.3
CDKN2C	NM_001262.2
CHEK2	NM_007194.4
CTNNA1	NM_001903.5
DLST	NM_001933.5
DNMT3A	NM_022552.4
EGLN1	NM_022051.2
EGLN2	NM_080732.3
ENG	NM_001114753.3
EPAS1	NM_001430.4
EPCAM *	NM_002354.3
ESR2	NM_001437.2
EXO1	NM_130398.4
FAN1	NM_014967.5
FH °	NM_000143.3
FLCN °	NM_144997.5
GALNT12	NM_024642.5
GCM2	NM_004752.3
GNA11	NM_002067.4
GOT2	NM_002080.4
GREM1 *	NM_013372.7
IDH3B	NM_006899.5

KIT	NM_000222.2
MAX	NM_002382.3
MDH2	NM_005918.3
MEN1	NM_130799.2
MET	NM_001127500.1
MLH1	NM_000249.3
MLH3	NM_001040108.2
MSH2	NM_000251.3
MSH3	NM_002439.5
MSH6	NM_000179.2
MUTYH	NM_001128425.1
MYO5B	NM_001080467.3
NF1	NM_000267.3
NTHL1	NM_002528.7
PBRM1	NM_018313.4
PDE11A	NM_0169853.3
PDE8B	NM_003719.3
PDGFRA	NM_006206.4
PMS2	NM_000535.7
POLD1 #	NM_002691.4
POLE #	NM_006231.4
PRKACA *	NM_002730.3
PRKAR1A	NM_002734.4
PTEN °	NM_000314.4
RET #	NM_020975.4
REXO2	NM_015523.4
RNF43	NM_017763.6
RPS20	NM_001146227.3
SCG5 *	NM_001144757.3
SDHA	NM_004168.3
SDHAF2	NM_017841.2
SDHB	NM_003000.2
SDHC	NM_003001.3
SDHD	NM_003002.3
SLC25A11	NM_003562.4
SMAD4	NM_005359.6
STK11	NM_000455.5
TGFBR2	NM_001024847.2
TMEM127	NM_017849.3
TP53 °	NM_000546.5
TSC1 °	NM_000368.4
TSC2 °	NM_000548.3
VHL °	NM_000551.3

*'limited to CNV
° evaluation of mosaicisms with allelic frequency >5%
RET extracellular cysteine rich domain ad tyrosine kinase domain [exons 5,8,10,11,13H16]
