# Supplementary material for: A novel pathogenic AIP variant associated with familial isolated pituitary adenoma
Source: Pituitary. 2026 Apr 20;29(3):75. doi: 10.1007/s11102-026-01672-y (PMC13095975; doi:10.1007/s11102-026-01672-y)
Supplement: Supplementary file 6 — Supplementary Material 6 (PDF 471 KB) [file 11102_2026_1672_MOESM6_ESM.pdf]

Pituitary

A novel pathogenic AIP variant associated with Familial Isolated Pituitary Adenoma

Valentino Marino Picciola[1*], Anna Crociara[2*], Serena Piacentini[3], Lucrezia Rossi[1], Maria Rosaria Ambrosio[1-2], Marco Gessi[4], Antonio d'Amati[4], Michele Rubini[5], Maria Chiara Zatelli[1-2]

* These Authors equally contributed to the work.

Affiliations

[1]Section of Endocrinology, Geriatrics and Internal Medicine, Department of Medical Sciences, University of Ferrara, 44124 Ferrara, ITALY

[2]Endocrine Unit, University Hospital S. Anna, 44124 Ferrara, ITALY

[3]Mater Olbia Hospital, Olbia, ITALY

[4]Department of Life Sciences and Public Health, Section of Anatomic Pathology, Università Cattolica del Sacro Cuore, Rome, Italy.

[5]Laboratory of Reproductive Medical Genetics, Department of Neuroscience and Rehabilitation, University of Ferrara, 44121 Ferrara, ITALY

Corresponding Author

Prof. Maria Chiara Zatelli

E-mail: ztlmch@unife.it

Supplementary Table 4: Clinical and biochemical features at first clinical evaluation in healthy family variant carriers.

**Subject**	**Age, Sex**	**Height** (cm)	**Weight** (kg)	**Acromegaly signs**	**IGF-1** (ng/ml)	**PRL** (5.18-26.5 ng/ml)	**Pituitary RM**
II-4	63, F	167	52	No	83,7 (38-244)	7,9	Negative
II-9	72, M	171	87	No	65,3 (25-242)	9,91	Negative
II-11	70, F	172	57	No	61,3 (26-226)	7,55	Negative
III-11	47, M	171	72	No	93,5 (71-224)	9,9	Negative
III-12	44, F	155	56	No	72 (69-253)	18,1	Negative
